# Green synthesis of silver nanoparticles using *Lysiloma acapulcensis* exhibit high-antimicrobial activity

**DOI:** 10.1038/s41598-020-69606-7

**Published:** 2020-07-30

**Authors:** Diana Garibo, Hugo A. Borbón-Nuñez, Jorge N. Díaz de León, Ernesto García Mendoza, Iván Estrada, Yanis Toledano-Magaña, Hugo Tiznado, Marcela Ovalle-Marroquin, Alicia G. Soto-Ramos, Alberto Blanco, José A. Rodríguez, Oscar A. Romo, Luis A. Chávez-Almazán, Arturo Susarrey-Arce

**Affiliations:** 1 Cátedras Conacyt-Centro de Investigación Científica y de Educación Superior de Ensenada (CICESE), Departamento de Microbiología, Ensenada, Baja California México; 2 Universidad Nacional Autónoma de México (UNAM), Centro de Nanociencias y Nanotecnología, Ensenada, Baja California México; 3 Cátedras Conacyt-Universidad Nacional Autónoma de México (UNAM), Centro de Nanociencias y Nanotecnología, Ensenada, México; 4 Centro de Investigación Científica y de Educación Superior de Ensenada (CICESE), Ensenada, Baja California México; 5 Cátedras Conacyt-Centro de Investigación en Materiales Avanzados S.C. (CIMAV), Departamento de Ingeniería de Materiales y Química, Chihuahua, México; 6 Universidad Autónoma de Baja California (UABC), Escuela de Ciencias de la Salud, Unidad Valle Dorado, Ensenada, México; 7 Agilent Technologies México, Ciudad de México, CDMX, México; 8 Secretaría de Salud de Guerrero, Banco de Sangre Regional Zona Centro, Chilpancingo de los Bravo, Guerrero México; 9 Mesoscale Chemical Systems, MESA+ Institute, University of Twente, Drienerlolaan 5, 7522 NB Enschede, The Netherlands

**Keywords:** Biomaterials, Antimicrobials

## Abstract

The scientific community is exploiting the use of silver nanoparticles (AgNPs) in nanomedicine and other AgNPs combination like with biomaterials to reduce microbial contamination. In the field of nanomedicine and biomaterials, AgNPs are used as an antimicrobial agent. One of the most effective approaches for the production of AgNPs is green synthesis. *Lysiloma acapulcensis* (*L. acapulcensis*) is a perennial tree used in traditional medicine in Mexico. This tree contains abundant antimicrobial compounds. In the context of antimicrobial activity, the use of *L. acapulcensis* extracts can reduce silver to AgNPs and enhance its antimicrobial activity. In this work, we demonstrate such antimicrobial activity effect employing green synthesized AgNPs with *L. acapulcensis*. The FTIR and LC–MS results showed the presence of chemical groups that could act as either (i) reducing agents stabilizing the AgNPs or (ii) antimicrobial capping agents enhancing antimicrobial properties of AgNPs. The synthesized AgNPs with *L. acapulcensis* were crystalline with a spherical and quasi-spherical shape with diameters from 1.2 to 62 nm with an average size diameter of 5 nm. The disk diffusion method shows the magnitude of the susceptibility over four pathogenic microorganisms of clinical interest. The antimicrobial potency obtained was as follows: *E. coli* ≥ *S. aureus* ≥ *P. aeruginosa* > *C. albicans*. The results showed that green synthesized (biogenic) AgNPs possess higher antimicrobial potency than chemically produced AgNPs. The obtained results confirm a more significant antimicrobial effect of the biogenic AgNPs maintaining low-cytotoxicity than the AgNPs produced chemically.

## Introduction

The field of material sciences encourages to obtain materials of various types of nanoscale shapes and architectures^1^.NPs with a size range of 1–100 nm, and different shapes provide unique chemical^2^, physical^3^ and optical properties^4,5^. NPs can be synthesized with physical, chemical and biological methods^6^. These methods might have unique advantages and disadvantages depending on the end application^7–11^. For example, physical methods might have some disadvantages when applied in microbiology. The methods can be time-consuming and constrain to specific requirements like high temperature or pressure, which might result unattractive owing to equipment and associated cost^12,13^. A key advantage of chemical methods is the accessibility to get the NPs in suspension. After synthesis and purification, the NPs can immediately be accessible for functionality testing^14^. However, in some cases, the synthesis procedure might result expensive owing to the material type used (e.g., borohydride, 2-mercaptoethanol, thioglycerol and citrate). For chemical synthesis methods applied in microbiology, the most critical point is the toxic effect of the NPs or by-products generated, especially when released to the environment^15^. On the contrary, biological methods (e.g., plant extract) utilize fewer toxic reactants and additives. The reaction can occur at room temperature without harsh or stringent reaction constraints. Plant extract can then provide low or no cytotoxicity when combined with NPs. Therefore, biological methods using plant extracts could be cataloged safe, eco-friendly and low-cost, representing a viable alternative when applied in microbiology.

Microorganisms (yeast, fungi, bacteria, viruses and actinomycete) and plant-mediated synthesis are different primary sources for NPs synthesis by biological methods^16^. Synthesized NPs using plant extracts are more advantageous than intracellular synthesis using microorganisms since it does not need complex and specialized processes such as isolation, culture maintenance and multiple purification steps. Due to the above, it has become a major focus leading researchers to develop green methods using different parts of the plant, e.g., from leaf^17–19^, peel^20^ flower^21^, fruit^22^ and root^23^. Many compounds present in the plant extract (e.g., p1olyphenols, ascorbic acids, flavonoids, terpenoids and proteins) play an essential role in the mechanism of metal ions uptake, reduction of precursor salt as well as capping agents and some of them with inherent antimicrobial properties.

*L. acapulcensis* is an endemic perennial tree of the south of Mexico, is abundant and has been empirically used in traditional medicine because of its properties, curative for respiratory, gastrointestinal, urinary and skin infections^24^. Few reports describe the chemical composition of *L. acapulcensis* extracts^25^. The extract has abundant tannin compounds which grant access to antimicrobial properties^26,27^. AgNPs are one of the most promising antimicrobial materials applied in nanomedicine. AgNPs can interact with the microorganism cell wall, generating reactive oxygen species that ultimately leads to cell death. Therefore, we can hypothesize that the use of *L. acapulcensis* extract can form AgNPs with improved antimicrobial activity. The combination of AgNPs with *L. acapulcensis* can then make a promising alternative against infectious diseases with reduced cytotoxicity.

In this work, we report the in vitro antimicrobial activity of AgNPs synthesized by a green method using a *L. acapulcensis* extract. The AgNPs were tested against three bacterial strains (*Escherichia coli*, *Pseudomonas aeruginosa* and *Staphylococcus aureus*) and one yeast (*Candida albicans*) of clinical interest. Morphology, size distribution, elemental analysis and electron diffraction pattern of nanoparticles were characterized. Minimum inhibitory concentration (MIC) and Minimum Biocidal Concentration (MBC) for the biogenic AgNPs were established and compared with those obtained with AgNPs synthesized by a chemical method. Less cytotoxic effects were found for biogenic AgNPs.

## Results

### Biophysical characterization

**Figure 1:**
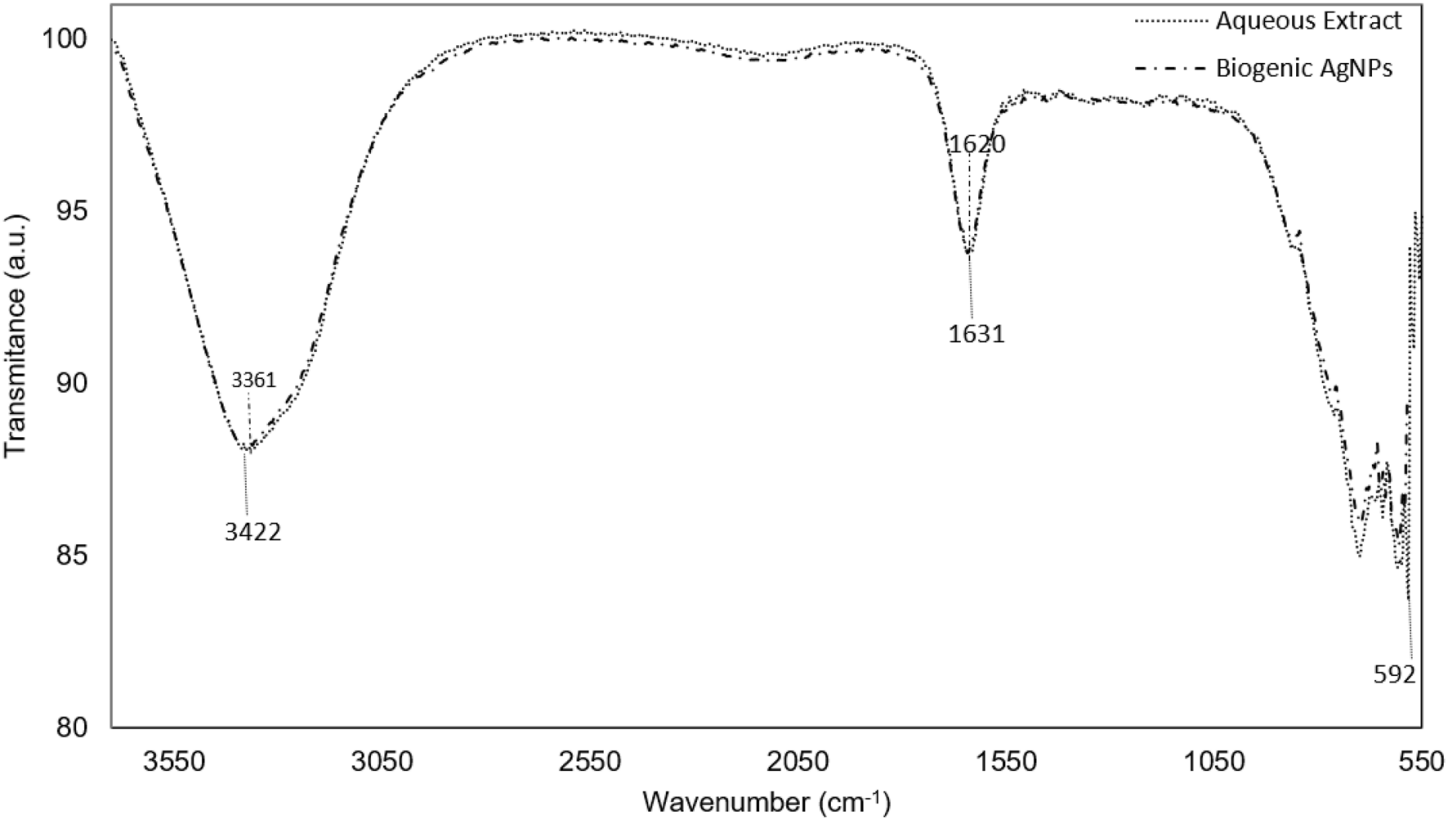
FTIR spectra of *L. acapulcensis* aqueous extract.

The FTIR spectra of the *L. acapulcensis* aqueous extract was recorded to identify functional groups. Their phytochemical profile revealed the presence of alkyl halides, proteins, phenolic and aromatic compounds with transmission peaks at 592, 1631, 2340 and 1620 cm^−1^, respectively (Fig. 1). The FTIR band around 3422 cm^−1^ is assigned to intramolecular H bonds, most probably from water molecules (Fig. 1). Moreover, LC–MS suggests the presence of different molecular components (Figure S1 & Table S1–S2) with either antimicrobial, surfactant and reducing character. Other molecular compounds were also identified to be part of the extract (Figure S1 & Table S1–S2).

**Figure 2:**
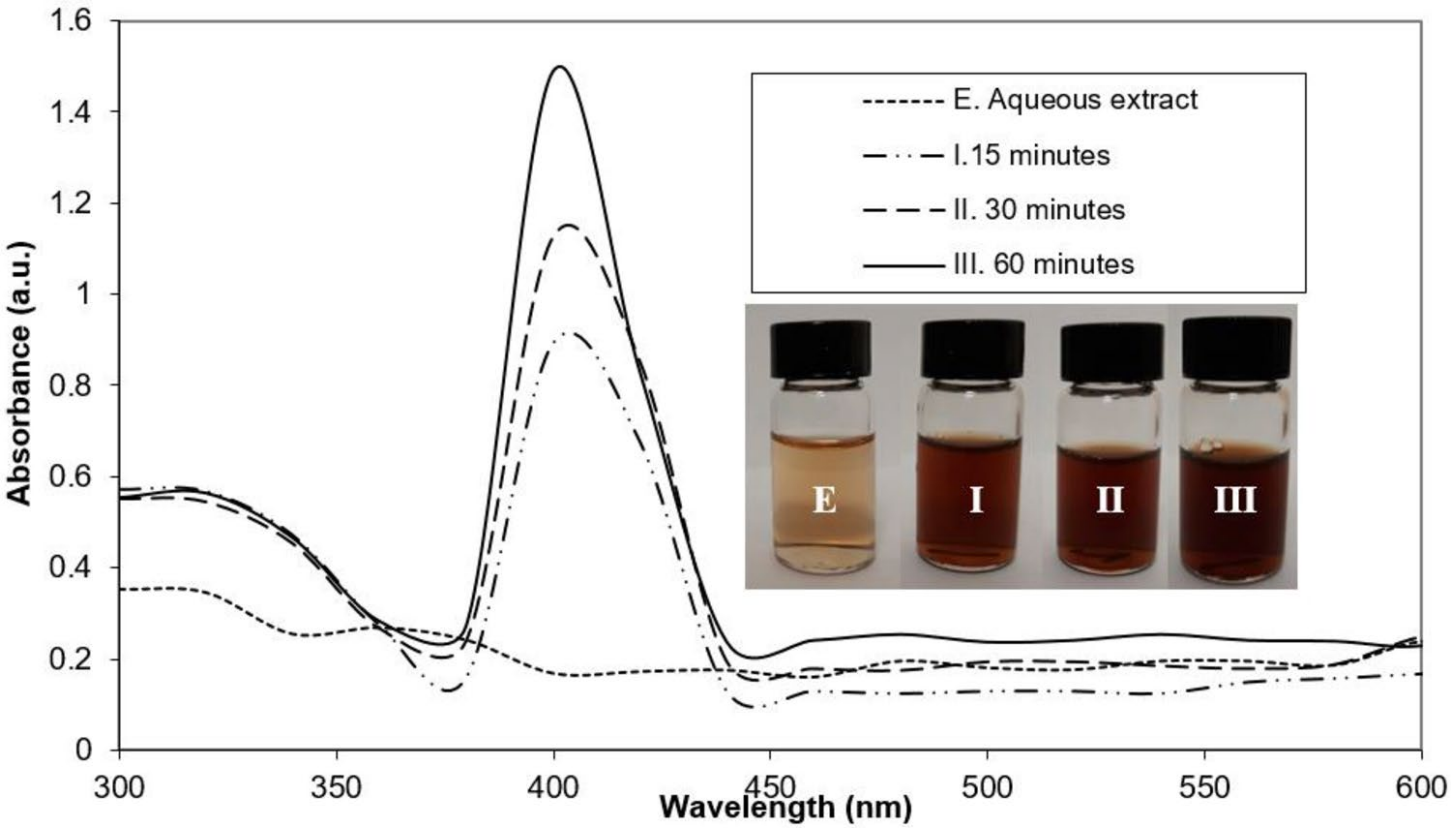
UV–vis absorption spectrum of biosynthesised silver nanoparticles using *L. acapulcensis* aqueous extract (E) with different incubation times (I.15, II. 30 and III. 60 min).

AgNPs were synthesized after the identification of the molecular structure and functional groups present in the *L. acapulcensis* extract. The AgNPs were first identified by a visual color change in the reaction mixture containing AgNO_3_ and *L. acapulcensis* extract over different incubation times (15, 30 and 60 min). Figure 2 shows the UV–Vis spectra (wavelengths ranging: 300–600 nm) recorded from each synthesized AgNPs. The color intensity increased with the duration of incubation time, turned from yellow to dark brown (Fig. 2). Biogenic AgNPs showed the maximum absorbance at 400 nm for solutions incubated for 15, 30 and 60 min, and the absorbance units increased in intensity (0.892, 1.125 and 1.488 a.u.) with time. No wavelength shift of the 400 nm peak was observed for the different reaction times. A color change of the suspension helped to determine AgNPs presence. Therefore, we use 15 min synthesis time for the experiments presented next.

### X-ray diffraction (XRD) analysis

**Figure 3:**
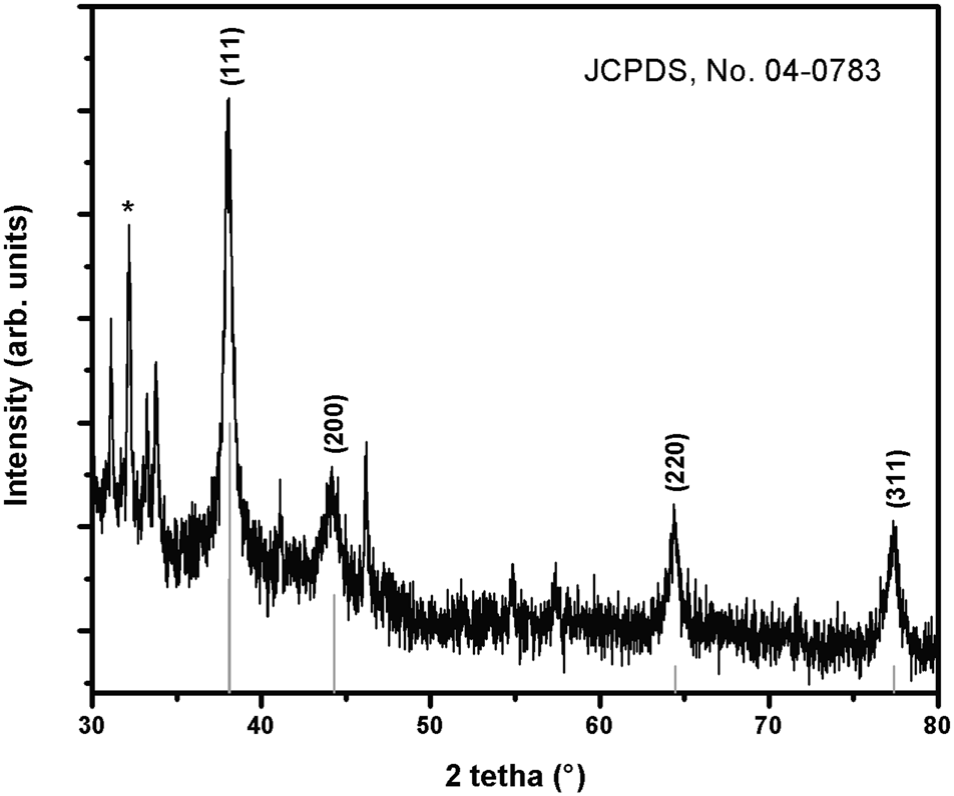
XRD pattern of biogenic Ag nanoparticles. Vertical lines correspond to face centered cubic (fcc) crystal structure of silver (JCPDS, No. 04-0783). (*cubic structure of AgK_3_, PDF 50–1435).

The crystalline nature of biogenic nanoparticles was confirmed by X-ray crystallography, recorded on a Panalitycal X’pert Pro MRD X-ray diffraction instrument, with Cu Kα radiation (λ = 0.15418 nm) over the scanning range 2θ = 30°–80°, with a step of 0.02 degree. The XRD pattern of the synthesized AgNPs (Fig. 3), shows several peaks, where the four main peaks located at 38.10°, 44.20°, 64.41° and 77.39°, corresponding to the (111), (200), (220) and (311) planes, respectively, to the facets of face-centered cubic (fcc) crystal structure of silver (JCPDS, No. 04-0783). An intense peak, located at 32.15° could be indexed to a cubic structure of AgK_3_ (PDF 50-1435). The presence of K was further confirmed during XPS and EDS experiments.

### TEM characterization of biogenic AgNPs

**Figure 4:**
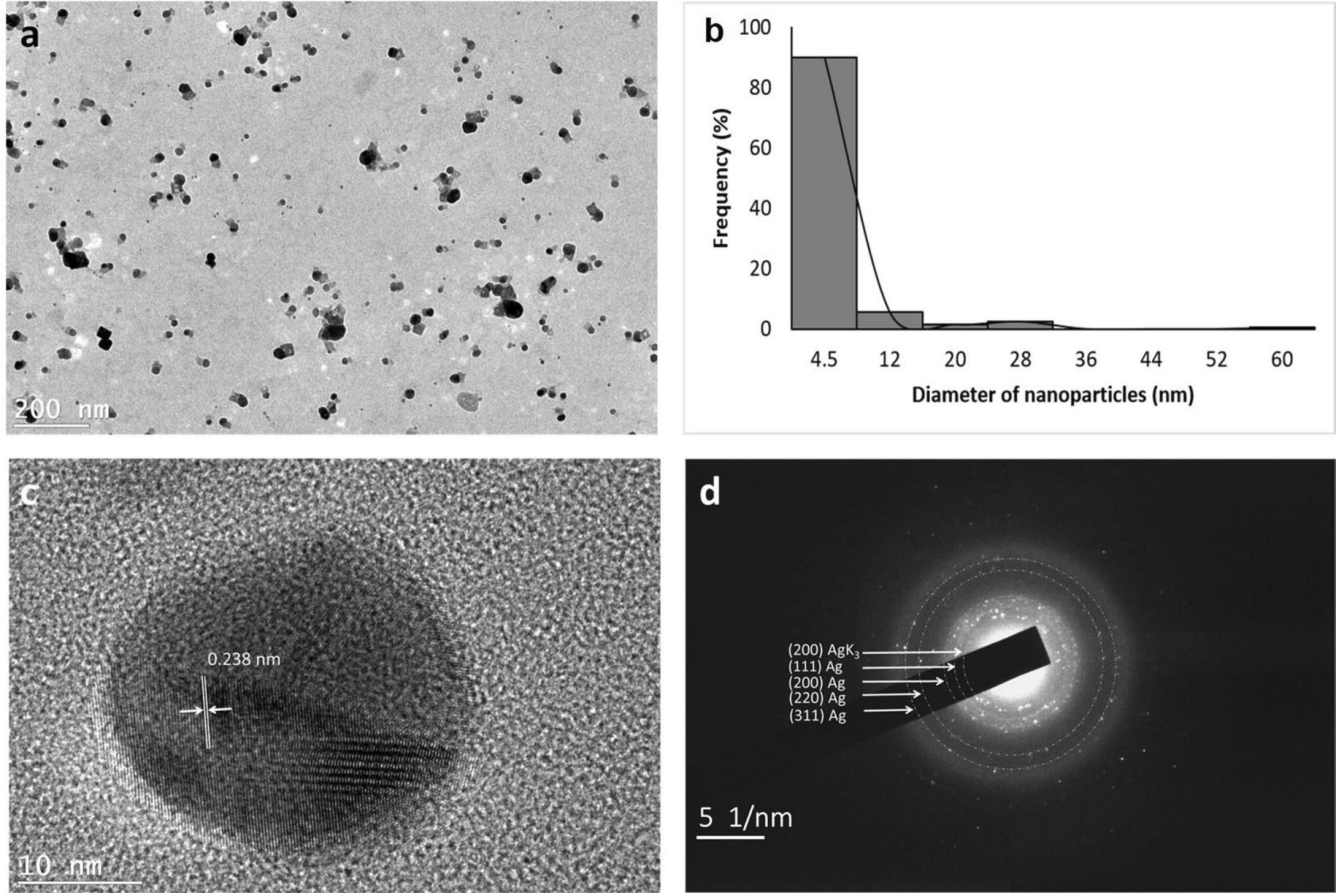
(**a**) Transmission electron microscopy (TEM) images of biogenic Ag nanoparticles (AgNPs). (**b**) Histogram of the particle diameter size distribution of the AgNPs. (**c**) High-resolution TEM image of individual Ag nanoparticle. (**d**) The selected area electron diffraction (SAED) pattern.

The morphology and size of biogenic AgNPs were analyzed by transmission electron microscopy (TEM). Figure 4a shows the presence of spherical and quasi-spherical nanoparticles. Their particle size distribution was obtained from a histogram, considering 200 particles, measured using JMicroVision version 1.2.7 (Fig. 4b). Biogenic nanoparticles shows a size range of 1.2–62 nm with an average size of 5 nm. High-resolution TEM (HRTEM) analysis was used to determine the structure of biogenic Ag nanoparticles (Fig. 4c). HRTEM shows the crystalline structure of single biogenic nanoparticle, with visible lattice fringes. A lattice spacing of 0.238 nm was calculated, corresponding to the plane family (111) of fcc silver. Additionally, Fig. 4d shows selected area electron diffraction (SAED) pattern, which indicates their polycrystalline nature and each of the diffraction rings has been indexed to (111), (200), (220) and (311), corresponding fcc crystal structure of metallic silver (JCPDS, No. 04-0783) and a diffraction ring corresponding to (200) cubic structure of AgK_3_ (PDF 50-1435). The results are consistent with the XRD diffractogram obtained in Fig. 3.

### Chemical characterization by XPS and EDS

**Figure 5:**
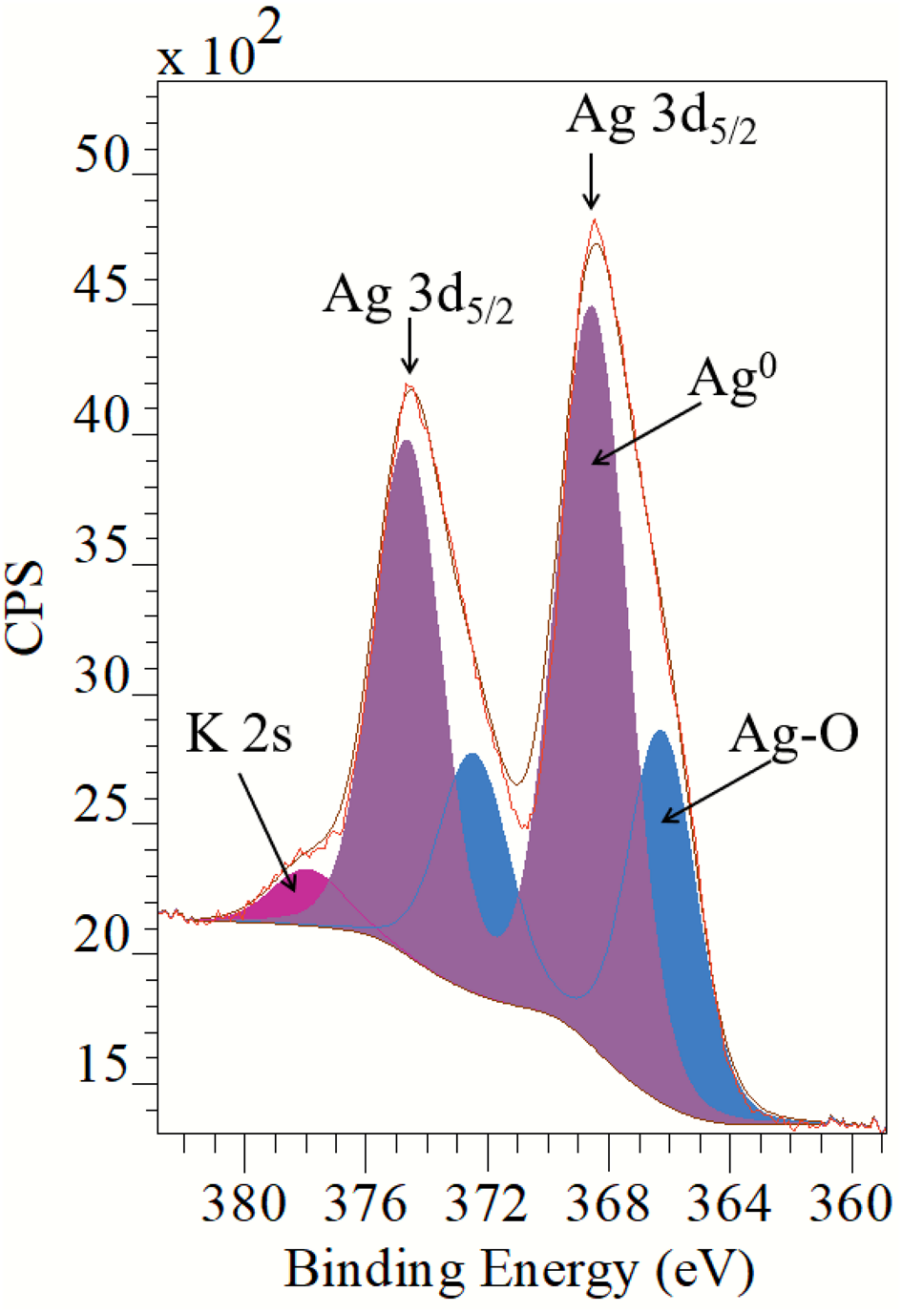
Detail decomposition of the AgNPS spectrum in the Ag 3d core emission region.

The general XPS survey showed the presence of C, O, N, Ag and K with traces of chlorine. A high-resolution spectrum was collected for the Ag 3d in Fig. 5. Our Gaussian–Lorentzian curves showed two chemical states of Ag. The curve assigned to Ag^0^ NPs resulted in 368.5 eV for the electrons arising from the Ag 3d_5/2_ level. AΔ = 6.0 eV was estimated between the Ag 3d_5/2_ and Ag 3d_3/2_. Then, the second chemical state is assigned to Ag–O (366.3 eV)^28^, with almost 2.2 eV, shifted to the lower binding energy when compared to Ag^0^. The presence of Ag–O can be related to Ag^+^ species, which might suggest the presence of Ag^+^ in suspension from unreacted Ag or AgNP leaching. In this region, we also observed the presence of the K 2s emission. The SEM–EDS chemical bulk quantification for the dry sample of NPs displayed the peaks related to the Ag, O, C and K clearly and some traces of P see supplementary information (Figure S2). Cl was not quantified by SEM–EDS analysis.

### Antimicrobial susceptibility testing

**Figure 6:**
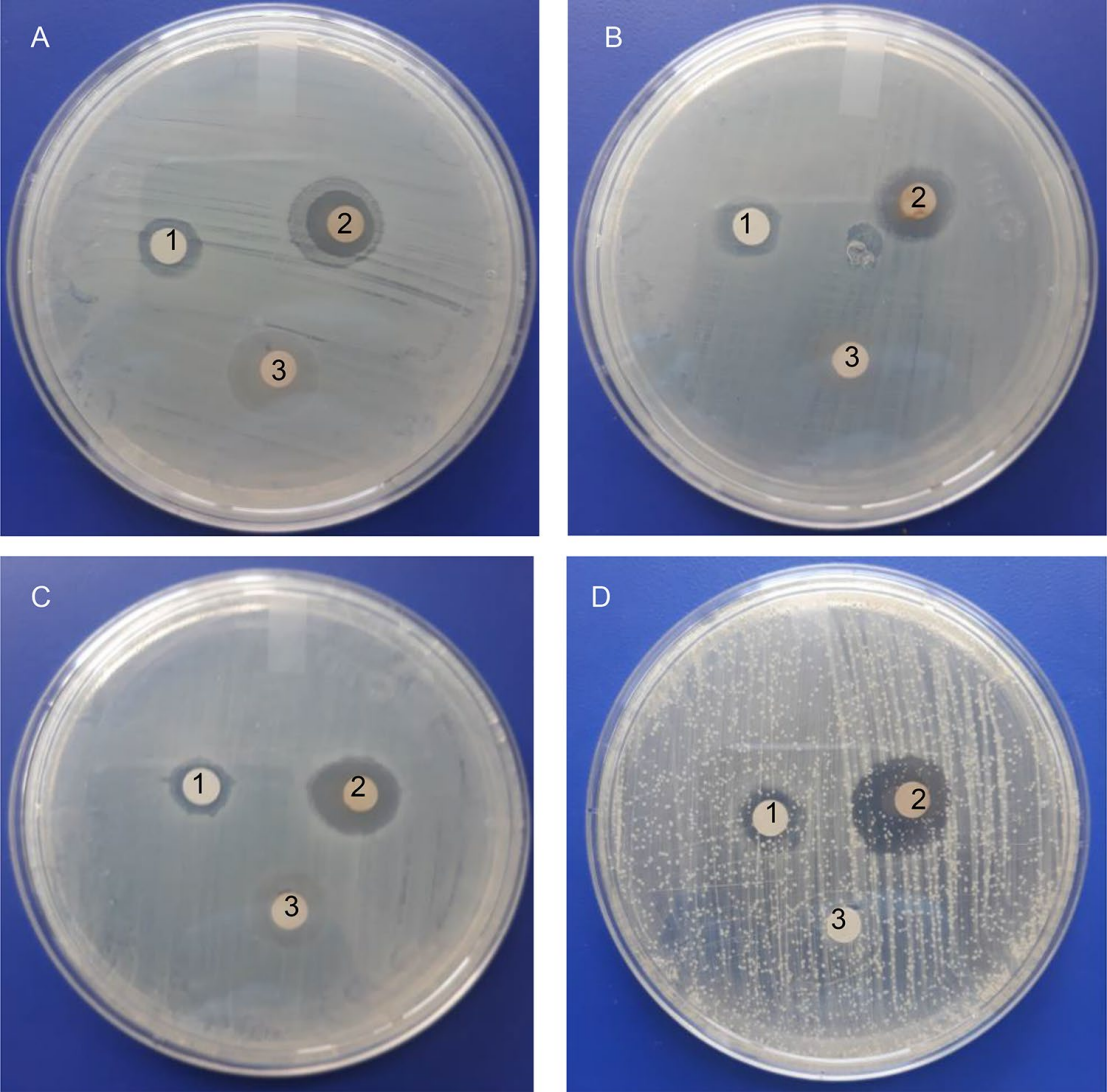
Antimicrobial susceptibility disk diffusion method. Zones of inhibition of chemical nanoparticles (1), biogenic nanoparticles (2) and aqueous extract (3) against the pathogenic strains *E. coli* (A), *P. aeruginosa* (B), *S. aureus* (C) and *C. albicans* (D).

The antimicrobial effect of biogenic AgNPs was investigated on four clinical pathogenic organisms (*E. coli*, *P. aeruginosa,* *S. aureus* and *C. albicans*) using the agar well diffusion method and by determining the MIC and MBC, the results were compared to those obtained by AgNPs synthesized by the chemical method. The disk diffusion method shows the magnitude of the susceptibility of the pathogenic microorganisms (Fig. 6). The aqueous extract produced a diffuse ring for all microorganisms. Biogenic AgNPs possessed higher antimicrobial activity than chemical AgNPs. The mean of three replicates of the diameter of inhibition zones (in millimeters) containing AgNPs suspension is presented in Table 1. Biogenic AgNPs showed a higher inhibitory effect against *C. albicans*. For this type of AgNPs, the inhibition zone reached 18.0 ± 1.3 mm for *E. coli*, 16.0 ± 1.0 mm for *S. aureus* and 15.0 ± 0.5 mm for *P. aeruginosa*. Lower inhibition zones were found for the chemical prepared AgNPs. The MIC and MBC were found between 2.5 µg/mL to 5.0 µg/mL for chemical NPs and between 0.06 and 0.25 µg/mL for biogenic NPs. These results confirm the high antimicrobial potency of biogenic NPs compared to chemical NPs (Table 1). The lowest MIC of biogenic NPs at 0.06 µg/mL was obtained against *E. coli*, *P. aeruginosa* and *S. aureus*. The growth of *C. albicans* inhibited at 0.13 µg/mL. MBC of chemical AgNPs were not recorded for all pathogenic microorganisms in the tested concentration range (≥5 µg/mL). Biogenic AgNPs inhibited at 0.25 µg/mL for *C. albicans* and 0.13 µg/mL for *E. coli*, *S. aureus* and *P. aeruginosa.*

**Table 1:** Mean zone of inhibition (mm) and antimicrobial activity (MIC and MBC values in µg/mL) of chemical nanoparticles (AgNPs _chemical_) and biogenic nanoparticles (AgNPs _*L.acapulcensis*_) against *E. coli*, *P. aeruginosa*, *S. aureus* and *C. albicans*.

Microorganism	Mean width of inhibition zone (mm)		MIC	MBC	MIC	MBC
	AgNPs_chemical_	AgNPs_*L.acapulcensis*_	AgNPs_chemical_		AgNPs_*L.acapulcensis*_	
*E. coli*	11 ± 0.6	18 ± 1.3	2.5	5	0.06	0.13
*P. aeruginosa*	11 ± 1.0	15 ± 0.5	5	>5	0.06	0.13
*S. aureus*	11 ± 0.5	16 ± 1.0	2.5	5	0.06	0.13
*C. albicans*	10 ± 0.1	19 ± 0.5	5	>5	0.13	0.25

The mean and standard deviation (SD) reported for each type of nanoparticle and with each microbial strain were based on three biological replicates. For MIC and MBC values, the standard deviation of a data set is zero because all of its values were identical.

### Cytotoxicity assay of biogenic AgNPs

**Figure 7:**
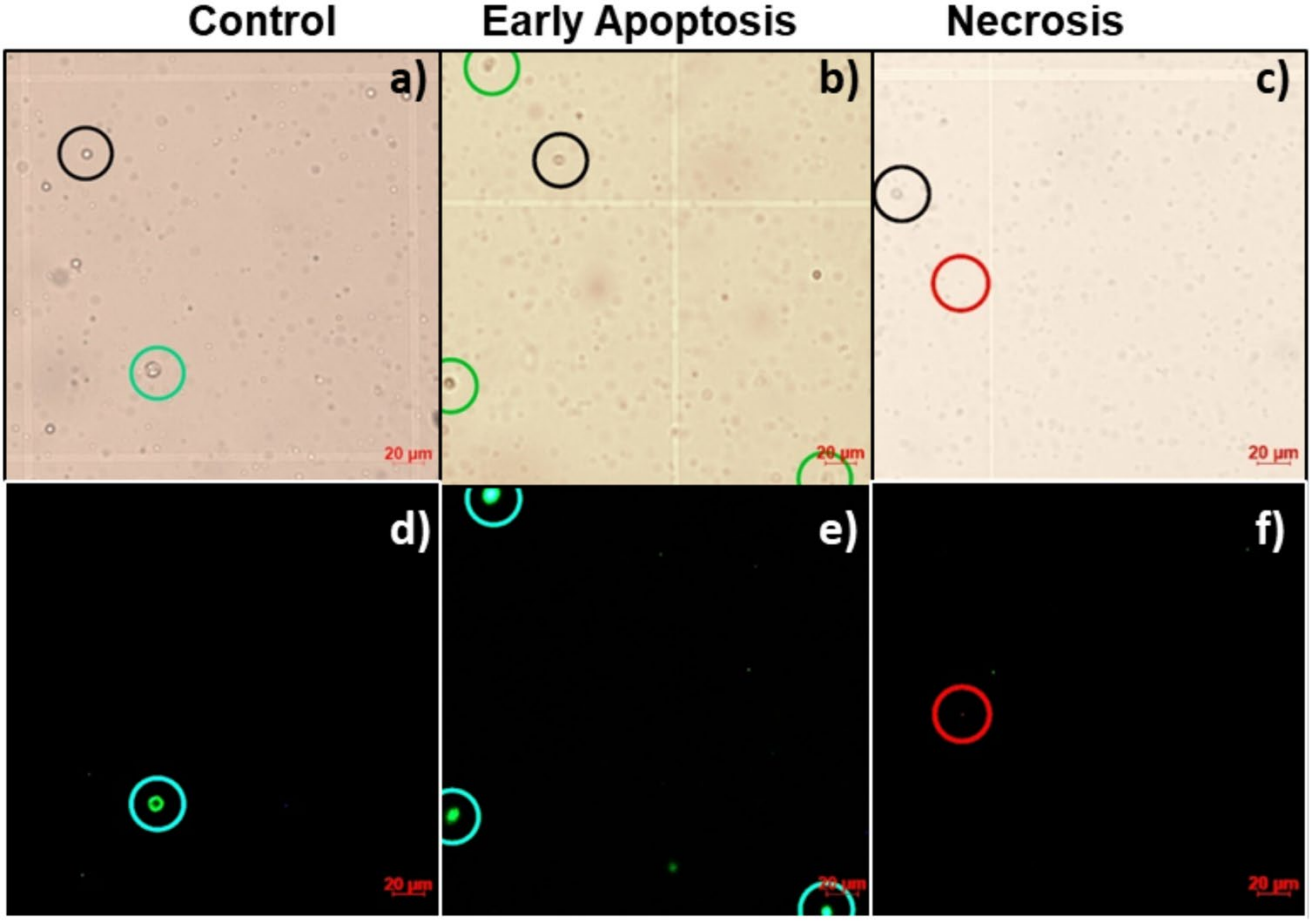
Micrographs of lymphocytes culture evaluated. Images show 40x  bright-field of control (**a**), and apoptotic (**b**) and necrotic (**c**) lymphocytes exposed to 1.3 µg/mL AgNPs. (**d**–**f**) show the corresponding image on fluorescent microscopy. Black, green and red circles show AV-/PI-, AV+/PI-stained lymphocytes and AV-/PI+ micronucleus.

Lymphocytes are an important part of the immune system and they have already been used to evaluate NPs cytotoxicity^29–31^. In this work, to determine the cytotoxic effect of biogenic AgNPs, human peripheral blood lymphocytes (HPBL) were treated with 1.3 µg/mL for 24 h. Viability was assayed by trypan blue exclusion test (Figure S3), no decrease in the viability of HPBL was observed. However, to determine if the biogenic AgNPs can lead to an earlier apoptosis death pathway, apoptosis/necrosis test was done using the Alexa fluor 488 cell death evaluation kit (Invitrogen, USA). Micrographs in Figure S3 show that exist more cells *AV*+ comparing to a negative control (untreated cells). This marker interacts with the phosphatidylserine in the plasma membrane, indicating an apoptotic cell death pathway. Moreover, no *PI*+ lymphocytes were found in the cultures treated (Fig. 7) that suggests these AgNPs are in an early stage of apoptosis that could be resolved by the cell, so these AgNPs are not harmful to the first line of immune defense of the organism.

## Discussion

This work shows the synthesis of biogenic AgNPs using an *L. acapulcensis* aqueous extract as a reducing agent for the first time. The *L. acapulcensis* extract has important biological components like proteins and ethylene groups detected by FTIR and could act as a capping/stabilization agents^32,33^,also, alkyl halides act as reducing agents. LC–MS reveals the presence of antimicrobial agents (18α-Glycyrrhetinic acid and ruspolinone) along with the presence of antioxidants (e.g., members of the azoles group) and alkaloids act as reducing agent (ruspolinone). These molecules and others can end-capping the AgNPs, enhancing AgNP antimicrobial activity greatly. Visual observation and UV–Vis spectroscopy easily followed the formation of biogenic AgNPs by reducing Ag^+^ ions into Ag^0^ ions. The surface quantification showed that Ag^0^ NPs resulted in 68% with 32% of Ag–O species. The Ag–O correspond with the remaining cations of the Ag precursor possibly from the unreacted Ag precursor during synthesis. It is important to note that leaching of Ag might play an important role, but the cytotoxicity assay was rather low for the green synthesized NPs. It was observed that upon the addition of the *L. acapulcensis* aqueous extract into the volumetric flask containing the AgNO_3_ solution, the color of the aqueous extract changed from yellow to dark brown within 15 min of incubation, this showed the formation of AgNPs. Its formation was also confirmed by the absorption peak at 400 nm in the UV–Vis spectra for all different incubation times. The intensity of brown color and absorbance units increases with the increase of the incubation period. Therefore, 15 min was used for the synthesis of biogenic AgNPs, providing an easy way and rapid protocol, which was an important advantage of biological methods compared to other methods using different primary biological material because they are normally synthesized within 30 min or more^21,22,34–37^. TEM showed a widespread distribution of biogenic AgNPs with spherical and quasi-spherical shapes. Some of them were elongated due to the aggregation of two or more nanoparticles. Their sizes were in the range 1.2–62 nm, with an average of 5 nm and 50% of the particles were in the 1 to 4 nm range. Similar results have been reported for AgNPs with sizes from 2 to 75 nm using plant extracts as reducing agents^34,35,38–41^.

There are studies and reports that plant extract NPs have strong antimicrobial activity. These AgNPs were effective in killing a range of bacterial pathogens involved in different infectious diseases, e.g., *S. aureus*, *P. aeruginosa*, *E. coli* and *C. albicans* are related to most common hospital-acquired infections^36,42^. Our biogenic AgNPs possessed higher antimicrobial activity than other biogenic AgNPs^40,41^. Reports on the mechanism of antimicrobial AgNPs action implies the dimorphic transition, DNA loses its ability to replicate and membrane disruption inhibiting bacterial growth^43^.

Navarro and collaborators reported the antimicrobial evaluation *L. acapulcensis* extracts with dichloromethane (D), hexane (H), water (W) and methanol (M). They used higher amounts of biomass in order to evidence the MIC values (*S. aureus*: 4.0 mg/mL (H), 1.0 mg/mL (D) and 1.0 mg/mL (M); *E. coli*: >8.0 mg/mL (H), 4.0 mg/mL (D) and 4.0 mg/mL (M); *C. albicans*: >8.0 mg/mL (H), 4.0 mg/mL (D) and 2.0 mg/mL (M)). Regarding the results of water extracts of *L. acapulcensis,* the authors did not mention why they did not present the MIC results^27^. In our case, the aqueous extract in the disk diffusion studies showed a diffuse ring, which is evidence of low antimicrobial activity. This low antimicrobial activity could be due to a lower amount of biomass (~3-fold lower than the biomass required by Navarro et al. ^26^ and Navarro et al. ^27^ used to obtain an aqueous extract of *L. acapulcensis*). The antimicrobial activity was enhanced using AgNPs synthesized from an aqueous extract of *L. acapulcensis* due to the combination with their antimicrobial substances present in the extract*.* Our MIC results were lower for *S. aureus, E. coli* and *C. albicans* than those obtained by Navarro and collaborators (*S. aureus*: 0.06 µg/mL *vs* 4.0 (H), 1.0 (D) and 1.0 mg/mL (M); *E. coli*: 0.06 µg/mL *vs* >8.0 (H), 4.0 (D) and 4.0 mg/mL (M) and *C. albicans*: 0.13 µg/mL *vs* >8.0 (H), 4.0 (D) and 2.0 mg/mL (M)) ^27^. For *P. aeruginosa*, a large zone of inhibition was compared to the one reported by Gopinath et al.^44^. Results obtained in the MIC and evidence of pronounced antibacterial activity of our biogenic AgNPs on *P. aeruginosa* can be compared to those obtained by other researches using different aqueous extract mediated AgNPs of different carnivorous plant tissue (*Drosera binata*, *Drosera indica*, *Drosera spatulata* and *Dionaea muscipula*)^45^. All the bacteria were eliminated with 0.13 µg/mL for *E. coli*, *S. aureus* and *P. aeruginosa* and 0.25 µg/mL for *C. albicans*. Several reports showed that lower MIC values had stronger antibacterial potencies, but their stability and biocompatibility are related to small sizes between 10 and 15 nm^46–48^. Additionally, the MIC and MBC values of biogenic AgNPs confirmed their greater antimicrobial potency than chemical AgNPs.

In short, we showed that it is possible to obtain biogenic AgNPs using an aqueous extract of *L. acapulcensis,* which provides a quick, efficient and simple technique for the green synthesis of nanomaterials. The presence of alkyl halides and other reducing agents in the extract of *L. acapulcensis* allows the reduction of Ag^+^ ions into AgNPs. The biosynthesized AgNPs showed a significant antimicrobial effect against *C. albicans*, *E. coli*, *S. aureus* and *P. aeruginosa*. Nevertheless, the microbicidal activity of biogenic AgNPs remains at lower concentrations than the chemically synthesized AgNPs. Our AgNPs did not induce a decrease in cell viability in human peripheral blood lymphocytes at the concentration and time evaluated. Prospective studies are needed to demonstrate the long-term efficacy and the potential beneficial impact on infectious diseases.

## Conclusions

The present work showed that it is possible to obtain biogenic AgNPs using an aqueous extract of *L. acapulcensis*. The biosynthesized AgNPs shows a significant antimicrobial effect against *C. albicans*, *E. coli*, *S. aureus* and *P. aeruginosa* at lower concentrations than chemically synthesized AgNPs. In terms of toxicity, biogenic AgNPs did not induce a decrease in cell viability in human peripheral blood lymphocytes.

## Methods

### Preparation of *L. acapulcensis* aqueous extract

The stem and roots of *L. acapulcensis* were dried in a laboratory oven at 60 °C for 2 h until constant weight was reached. Afterward, 2 g of biological material were immersed in distilled water (100 mL) and were kept in a heated plate and allowed to boil for 15 min. The extract was filtered and kept refrigerated at 4 °C.

### HPLC-ESI-QTOF-MS analysis of *L. acapulcensis* extract

The plant extract was filtered through 0.2 µm PVDF filters (Sterivex, Millipore, Bedford, MA, USA). Determination of phytochemical composition was performed on a UHPLC (model 1260) coupled to a 6530 model Accurate-Mass QTOF LC/MS; Agilent Technologies (Palo Alto, CA, USA) equipped with an ESI interface operating in positive ion mode and an Agilent XDB-C8 2.7 μm 3 × 50 mm, 2.7 column. The mobile phase was 0.2% formic acid in water as eluent A and 0.1% formic acid in acetonitrile as eluent B, with the following set of operation parameters: Capillary voltage, 3500 V; nebulizer pressure, 35 psi; dry gas flow, 8l/min; dry gas temperature, 300 °C ; LC–MS mass spectra were recorded across the range mass 100–1,700 m/z; fragmentor 135 V; column temperature 40 ^o^C; solvent gradient conditions: 0 min, 0% B; 5 min, 10% B; 10 min, 80% B; 12 min, 100% B and then 15 min, 0%B. Compound identification was performed through MassHunter Workstation software using libraries G3874-60007 Massahunter METLIN PCDL B.08.00.

### Synthesis of biogenic nanoparticles

AgNPs were synthesised by reducing a silver nitrate solution at 0.001 M in the presence of the *L. acapulcensis* extract. A volume of 2.5 mL of AgNO_3_ was added to 2.5 mL of *L. acapulcensis* aqueous extract (ratio 1:1) and incubated at ambient conditions for 2 min. Then the reaction solution was prepared to a final volume of 10 mL with distilled water and the solutions were exposed under white light during 15, 30 and 60 min. The synthesis progress was monitored using UV–Vis spectroscopy (JENWAY, model 6505, UK) with a wavelength range from 300 to 600 with a resolution of 1 nm. The aqueous extract was used as a blank. Experiments were carried out in triplicate. FTIR spectroscopy analysis was carried out to reveal the functional group of biomolecules present in the *L. acapulcensis* aqueous extract using the instrument BRUKER Tensor 27 at room temperature with a range of resolution of 400–4000 cm^−1^.

### Synthesis of AgNPs by chemical method

For the synthesis of AgNPs by a chemical method, silver nitrate solution (0.001 M) and sodium borohydride (0.1 M) were used as a metal salt precursor and a reducing agent, respectively. Polyethylene glycol (PEG) 80 at 50 mM was used as a stabilizing agent.

### Characterization of AgNPs

The strutural characterization of biogenic AgNPs was analized by X-ray difraction, recorded on a Panalitycal X’pert Pro MRD X-ray diffraction instrument, with Cu Kα radiation (λ = 0.15418 nm) over the scanning range 2θ = 30–80°, with a step of 0.02 degree. The synthesized biogenic AgNPs were examined on a Hitachi H-7500, JEOL 2010 and a HRTEM, JEM-2200FS, JEOL, transmission electron microscopes. Samples were prepared by placing a 10 µL aliquot of biogenic AgNPs on carbon-coated copper grids (300 mesh, Ted Pella Inc.). At least 200 particles were measured using the software JMicroVision version 1.2.7 (www.jmicrovision.com) to characterize the size distribution of AgNPs. The SEM–EDS chemical bulk analysis was carried in a High-resolution SEM microscope from JEOL model JSM-S300 equipped with an energy dispersive X-ray system at 35 kV for 100 s. The sample was placed in a carbon doble-face tape. Surface analysis of the the AgNPs material was carried out by XPS using a commercial instrument SPECS spectrometer equipped with a PHOIBOS® 150 WAL hemispherical electron analyzer and an AlKα X-ray source. The sample was placed in a molybdenum holder with a micro indium foil to avoid adventitious carbon. Gaussian–Lorentzian curve fitting was done using the CasaXPS software Ver. 2.3.23 in the high-resolution emission-line regions. Semi-quantitative analysis of the chemical states was calculated for each contribution based on its peak area percentages.

### Antimicrobial sensitivity testing

The microorganisms used for antimicrobial susceptibility evaluation were: *E. coli* (ATCC 25922), *P. aeruginosa* (ATCC 27853), *S. aureus* (ATCC 49476) and *C. Albicans* (ATCC 49476). The disk diffusion method was used to determine a zone of inhibition. Aliquots of 0.1 mL of each test organism were spread on LB agar for bacteria and PDA agar for *C. albicans*. Each Petri plates were dried. Paper discs loaded with 10 µL of biogenic AgNPs, chemical AgNPs and *L. acapulcensis* aqueous extract were placed on the surface of agar plates and incubated at 37 °C during 24 h after which diameters of inhibition zones were measured. All the tests were run in triplicate and the average result was taken.

### Determination of minimum inhibitory concentration and minimum biocidal concentration

The Minimal Inhibitory Concentration (MIC) and Minimum Biocidal Concentration (MBC) were determined using the agar dilution method. Inoculums of bacteria (1–1.5 × 10^8^ CFU/ml) and *C. albicans* (1–5 × 10^6^ CFU/ml) were prepared according to the 0.5 McFarland standard. Stock solutions of AgNPs were two-fold concentrated for bacteria and ten-fold concentrated for yeast in concentrations in the range of 0.1–5 µg/mL for chemical nanoparticles and 0.02–1 µg/mL for biogenic nanoparticles. Then 0.5 mL of treatment (bacteria or yeast) and 0.5 mL of AgNPs were mixed and incubated at 37 °C during 24 h at 250 rpm (Orbit Environ Shaker). After incubation, 10 μL of each tube were transferred to LB agar for bacteria and YPD agar for *C. albicans* and incubated at 37 °C for 24 h. The MIC was the lowest concentration of an antimicrobial that inhibits the visible growth and the MBC was the lowest concentration that killed ≥ 99.9% of cells. The MIC and MBC were determined in triplicate and were carried out on at least three different days. Positive control was used for each microorganism (without treatment).

### Isolation of human peripheral blood lymphocytes

Human Peripheral Blood Lymphocytes (HPBL) were isolated from a blood sample obtained by venipuncture of a healthy donor using heparinized tubes. Blood samples were collected from healthy donors with prior informed consent. Protocols to collect and disposed blood samples were according to the mexican normativity (NOM-253-SSA1-2012, NOM-003-SSA1-1993) and approved by the Ethical Committee of the Health Sciences School from Autonomous University of Baja California, Mexico with file number 003/2019. Blood was first diluted 1:1 (v/v) in physiological saline solution and subsequently separated by ficoll density gradient centrifugation^49^, consistent with the providers protocol (Ficoll® Paque Plus GE Healthcare, USA). Briefly, 12 mL of diluted blood was carefully placed over 2 mL of ficoll, then centrifuged at 400G during 30 min. The layer of mononuclear cells was transferred to a centrifuge tube under sterile conditions. Cells were washed with physiological saline solution and seeded on a petri dish with RPMI 1640 media supplemented with 10% of autologous serum and incubated 2 h at 37 °C with 70% of relative humidity and 5% CO_2_. After the incubation time, lymphocytes (non-adherent cells) were taken from the supernatant and monocytes (adherent cells) were discarded.

### Cell viability assay

Lymphocytes isolated from human peripheral blood were used for cytotoxicity determination. 5 × 10^5^ lymphocytes per well were seeded in 96 well tissue culture plate and subsequently treated with AgNPs at a final concentration of 1.3 µg/mL. Cells were incubated for 24 h at 37 °C and 5% CO_2_. Viability was determined by the Trypan blue exclusion test using a Neubauer’s chamber under optical microscope. Untreated cells were used as negative control and 3 experiments by triplicate were done.

### Apoptosis and necrosis determination

Cells were obtained and treated as for the cell viability assay. In this case, after incubation, cells were stained using the Alexa Fluor™ 488 kit (Invitrogen, USA) according to the providers protocol. Briefly, cells treated and controls placed in a 96 well plate were added with 20 µL of binding buffer 1x (BD Pharmingen™, USA) and 1 µL Anexin V, incubated 15 min and stained whit 1 µL of Propidium lodide followed by 15 more min of incubation under a 5% CO_2_ atmosphere at 37 °C to identify apoptotic and necrotic cells. Samples were then observed through a 40x  objective using AxioCam ICc5 on 0.63 × C-Mount 60 N-C interface camera using a fluorescence microscope (Axio Lab A1, Carl ZEISS) for live cellular imaging. Excitation wavelengths of 455 nm were used to excite AV and PI.

### Data analysis

All the measurements were replicated three times for each assay and the results are presented as mean ± SD. Data were analyzed using Minitab version 18.0 and the descriptive statistic was used.

## Supplementary information

Supplementary information
