# Supplementary material for: Green synthesis of silver nanoparticles using Lysiloma acapulcensis exhibit high-antimicrobial activity
Source: Sci Rep. 2020 Jul 30;10:12805. doi: 10.1038/s41598-020-69606-7 (PMC7393152; doi:10.1038/s41598-020-69606-7)
Supplement: Supplementary file 1 — Supplementary information [file 41598_2020_69606_MOESM1_ESM.pdf]

Supplementary Information

Green synthesis of silver nanoparticles using *Lysiloma acapulcensis* exhibit high-antimicrobial activity

Diana Garibo[1,2*], Hugo A. Borbón-Nuñez[3], Jorge N. Díaz de León[2], Ernesto García Mendoza[4], Iván Estrada[5], Yanis Toledano-Magaña[6], Hugo Tiznado[2], Marcela Ovalle-Marroquin[4], Alicia G. Soto-Ramos[7], Alberto Blanco[2], José A. Rodríguez[2], Oscar A. Romo[2], Luis A. Chávez-Almazán[8], Arturo Susarrey-Arce[9]

[1]Cátedras Conacyt- Centro de Investigación Científica y de Educación Superior de Ensenada (CICESE), Departamento de Microbiología, Ensenada, Baja California, México.

[2]Universidad Nacional Autónoma de México (UNAM), Centro de Nanociencias y Nanotecnología, Ensenada, Baja California.

[3]Cátedras Conacyt-Universidad Nacional Autónoma de México (UNAM), Centro de Nanociencias y Nanotecnología, Ensenada, México.

[4]Centro de Investigación Científica y de Educación Superior de Ensenada (CICESE), Ensenada, Baja California, México.

[5]Cátedras Conacyt-Centro de Investigación en Materiales Avanzados S.C. (CIMAV), Departamento de Ingeniería de Materiales y Química, Chihuahua, México.

[6]Universidad Autónoma de Baja California (UABC), Escuela de Ciencias de la Salud, Unidad Valle Dorado

[7]Agilent Technologies México, Ciudad de México, CDMX

[8]Secretaria de Salud de Guerrero, Banco de Sangre Regional Zona Centro, Chilpancingo de los Bravo, Gro.

[9]Mesoscale Chemical Systems, MESA+ Institute, University of Twente, Drienerlolaan 5, 7522 NB, Enschede, the Netherlands

***dgaribo@conacyt.mx**

Supplemental Material

1. Liquid chromatography–mass spectrometry

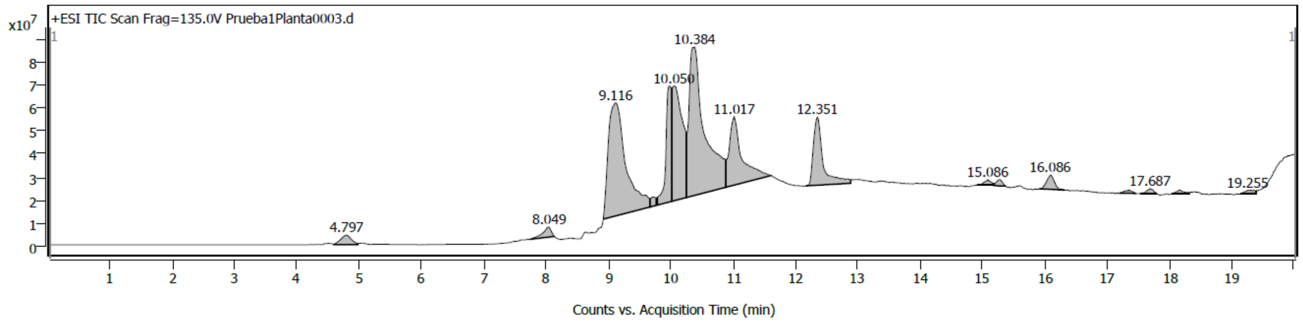

Figure S1. Liquid chromatography of the *L. acapulcensis* extract. The retention time (RT) of the identified components is presented in supplementary Table 1.

Supplementary Table 1. RT of the identified from the *L. acapulcensis* extract.

Peak	RT	Height	Area	Area%
1	4.797	4061622	49051488	4.25
2	8.049	4293531	41522177	3.6
3	9.116	49028434	9.64E+08	83.56
4	10.05	49714935	5.71E+08	49.48
5	10.384	64236008	1.15E+09	100
6	11.017	29627756	4.31E+08	37.32
7	12.351	29488946	3.23E+08	28.02
8	15.086	2396807	17202125	1.49
9	16.086	6139521	53672255	4.65
10	17.687	2165451	15438154	1.34
11	19.255	1421337	14810311	1.28

Supplementary Table 2. Spectrometric data of compounds found in the *L. acapulcensis* extract from Figure S1 is presented.

Peak No.	M. Cal. (g/mol)	M (m/z) found	Formula	Compound name	Effect*	Ref
1	100.12	100.05	$C_5H_8O_2$	2,3-pentanedione	**S**	[1]
2	212.14	213.38	$C_{12}H_{21}NS$ ion (M+H)+	2-heptyl-4,5-dimethyl-1,3-thiazole	**S, R**	[2]
3	470.68	471.34	$C_{30}H_{46}O_4$ (M+H)+	18α-Glycyrrhetinic acid	**A**	[3]
4-9	474.7	475.3	$C_{32}H_{42}O_3$ (M+H)+	1α-hydroxy-23-[3-(1-hydroxy-1-methylethyl)phenyl]-22,22,23,23-tetradehydro-24,25,26,27-tetranorvitamin D3 / 1α-hydroxy-23-[3-(1-hydroxy-1-methylethyl)phenyl]-22,22,23,23-tetradehydro-24,25,26,27-tetranorcholecalciferol	**S**, **PE**	[4]
10	314.33	337.10	$C_{14}H_{14}N_6O_3$ (M+Na)+	Dihydropteroic acid	**O**	[5]

11	262.97	267.16	$C_{14}H_{19}N_2O_3$ (M+H)+	Ruspolinone	**R, A**	[6]

***S** – possibly stabilizing agent of AgNPs; **R** – possibly reducing agent of $AgNO_3$; **A** -Antimicrobial activity; **PE** – known plant extract; **O** – others

2. SEM-EDS chemical bulk quantification for the dry sample of biogenic NPs

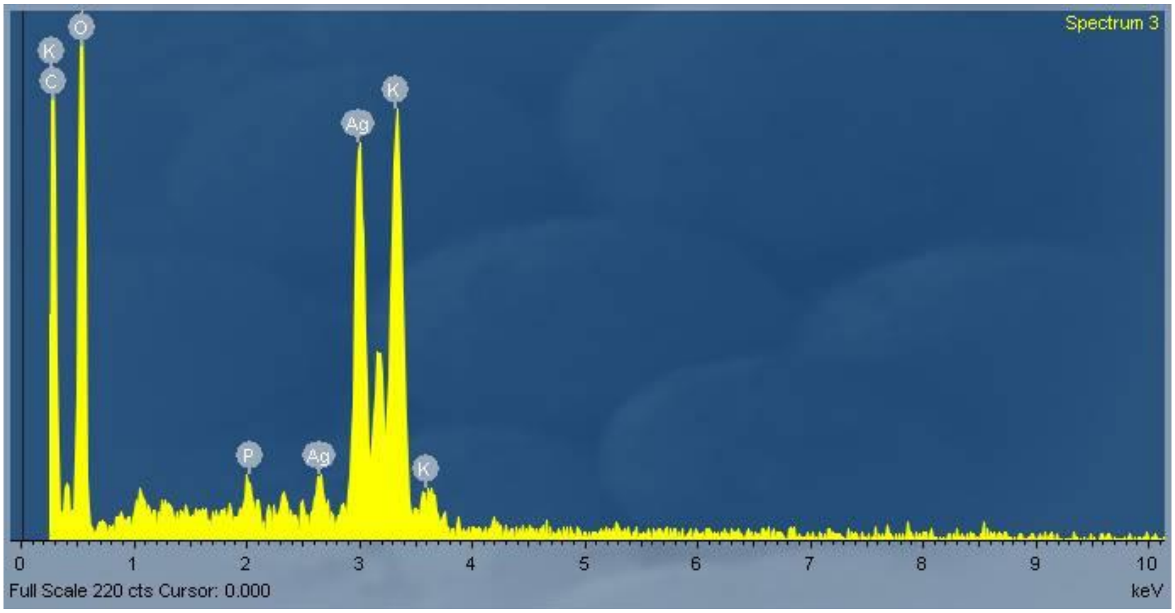

Figure S2. SEM-EDS chemical analysis of the AgNPs.

3. Cytotoxicity assay

Lymphocytes of human peripheral blood isolated by ficoll centrifugation gradient were used to determine total live and dead cells after 24 hours of exposure to 1.3 µg/ml. AV/PI were used to stained lymphocytes to determine death cell mechanism.

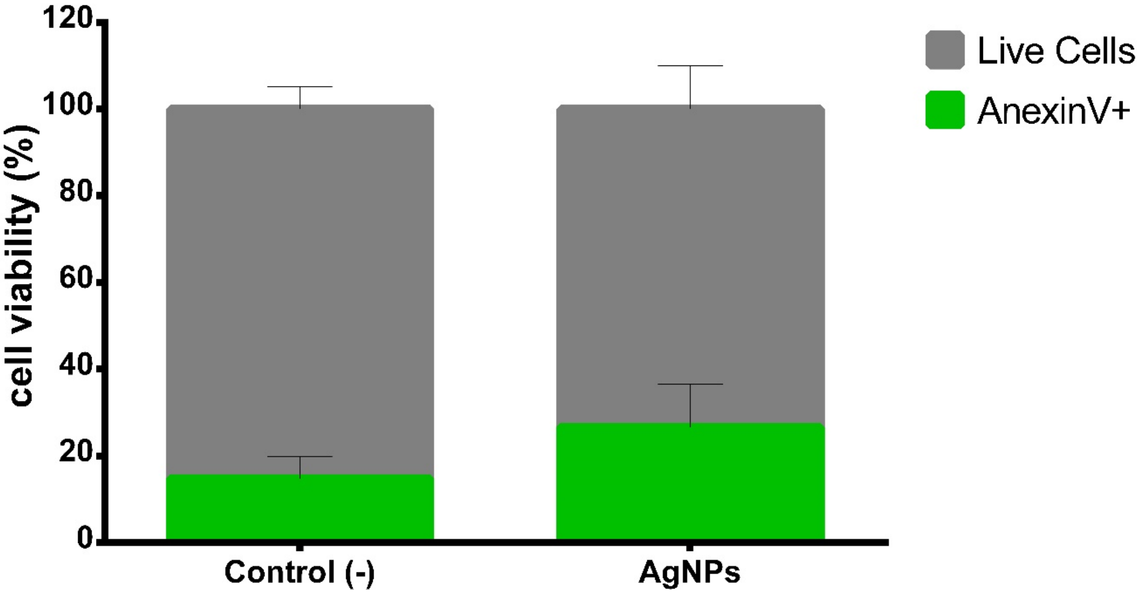

Figure S3. Cellular viability of lymphocytes evaluated. We show percentile values of cellular count for AV-/PI- (live cells, gray) and AV+/PI- (AnexinV+, green). Control (-) are untreated cells

References

[1] Oluwaseun, R.A., Nour, H.A., Chinonso, I.U. & Nassereldeen, A.K. Extraction and characterization of bioactive compounds in *Vernonia amygdalina* leaf ethanolic extract comparing Soxhlet and microwave-assisted extraction techniques. J Taibah Univ Sci **13**, 414-422, DOI: 10.1080/16583655.2019.1582460 (2019).

[2] Shmuel, Y. Dictionary of Food Compounds with CD-ROM (ed. Shmuel, Y.)1784 (2003), https://doi.org/10.1201/9781420068450.

[3] Kowalska, A. & Kalinowska-Lis, U. 18β-Glycyrrhetinic acid: its core biological properties and dermatological applications. Int. J. Cosmet. Sci. **41**, 325-331, https://doi.org/10.1111/ics.12548 (2019).

[4] Ziani, K., Fang, Y. & McClements, D.J. Encapsulation of functional lipophilic components in surfactant-based colloidal delivery systems: Vitamin E, vitamin D, and lemon oil. Food Chem **134**, 1106-1112, https://doi.org/10.1016/j.foodchem.2012.03.027 (2012).

[5] Sharma, S. & Anand, N. Chapter 18 – Antifolates. Food Chem **25**, 439-454, https://doi.org/10.1016/S0165-7208(97)80040-2 (1997)

[6] Eze, P.M. *et al*. Screening of metabolites from endophytic fungi of some Nigerian medicinal plants for antimicrobial activities. Pharm Biotech **3**, 9-18, DOI: 10.2478/ebtj-2019-0002 (2019).
